# Long-term nusinersen treatment across a wide spectrum of spinal muscular atrophy severity: a real-world experience

**DOI:** 10.1186/s13023-023-02769-4

**Published:** 2023-08-04

**Authors:** Anna Łusakowska, Adrianna Wójcik, Anna Frączek, Karolina Aragon-Gawińska, Anna Potulska-Chromik, Paweł Baranowski, Ryszard Nowak, Grzegorz Rosiak, Krzysztof Milczarek, Dariusz Konecki, Zuzanna Gierlak-Wójcicka, Małgorzata Burlewicz, Anna Kostera-Pruszczyk

**Affiliations:** 1 Department of Neurology, Medical University of Warsaw, ERN EURO-NMD, ul. Banacha 1a, Warsaw, 02-097 Poland; 2 Department of Neurology and Stroke, Ludwik Rydygier Specialist Hospital, Osiedle Złotej Jesieni 1, Kraków, 31-826 Poland; 3 Department of Econometrics, Faculty of Economics and Sociology, University of Łódź, ul. Rewolucji 1905 37/39, Łódź, 90-214 Poland; 4 Department of Radiology, Medical University of Warsaw, ul. Banacha 1a, Warsaw, 02-097 Poland

**Keywords:** Patient global impression – improvement, Computed tomography–guided lumbar puncture, Functional tests, Nusinersen, Scoliosis, *SMN2* gene, Spinal muscular atrophy

## Abstract

**Background:**

Spinal muscular atrophy (SMA) is an autosomal recessive disorder caused by a biallelic mutation in the *SMN1* gene, resulting in progressive muscle weakness and atrophy. Nusinersen is the first disease-modifying drug for all SMA types. We report on effectiveness and safety data from 120 adults and older children with SMA types 1c-3 treated with nusinersen.

**Methods:**

Patients were evaluated with the Hammersmith Functional Motor Scale Expanded (HFMSE; n = 73) or the Children’s Hospital of Philadelphia Infant Test of Neuromuscular Disorders (CHOP-INTEND; n = 47). Additionally, the Revised Upper Limb Module (RULM) and 6-minute walk test (6MWT) were used in a subset of patients. Patients were followed for up to 30 months of nusinersen treatment (mean, SD; 23, 14 months). Subjective treatment outcomes were evaluated with the Patients Global Impression–Improvement (PGI-I) scale used in all patients or caregivers at each follow-up visit.

**Results:**

An increase in the mean HFMSE score was noted at month 14 (T14) (3.9 points, p < 0.001) and month 30 (T30) (5.1 points, p < 0.001). The mean RULM score increased by 0.79 points at T14 (p = 0.001) and 1.96 points (p < 0.001) at month 30 (T30). The mean CHOP-INTEND increased by 3.6 points at T14 (p < 0.001) and 5.6 points at month 26 (p < 0.001). The mean 6MWT improved by 16.6 m at T14 and 27 m at T30 vs. baseline. A clinically meaningful improvement in HFMSE (≥ 3 points) was seen in 62% of patients at T14, and in 71% at T30; in CHOP INTEND (≥ 4 points), in 58% of patients at T14 and in 80% at T30; in RULM (≥ 2 points), in 26.6% of patients at T14 and in 43.5% at T30; and in 6MWT (≥ 30-meter increase), in 26% of patients at T14 and in 50% at T30. Improved PGI-I scores were reported for 75% of patients at T14 and 85% at T30; none of the patients reporting worsening at T30. Adverse events were mild and related to lumbar puncture.

**Conclusions:**

In our study, nusinersen led to continuous functional improvement over 30-month follow-up and was well tolerated by adults and older children with a wide spectrum of SMA severity.

**Supplementary Information:**

The online version contains supplementary material available at 10.1186/s13023-023-02769-4.

## Background

Spinal muscular atrophy (SMA) is an autosomal recessive disorder caused by a biallelic mutation in the survival motor neuron gene *SMN1* on chromosome 5q13 [1–3]. The lack of the SMN protein leads to anterior horn cell degeneration in the spinal cord, resulting in progressive muscle weakness and atrophy [4]. The *SMN2* gene is a centromeric copy of the *SMN1* gene, but the genes differ by a C-to-T transition in exon 7. This difference results in the exclusion of exon 7 during the *SMN2* pre–messenger-RNA splicing and production of the nonfunctional SMN protein, with only 10–15% of the *SMN2* product being a full-length protein [5, 6]. The number of the *SMN2* copies is the most important known modifier of SMA severity [7].

The incidence of SMA is about 1:11,000, and the carrier frequency is 1 in 40 to 67 [8]. The phenotype of SMA ranges from a severe infantile form, with hypotonia and generalized weakness at birth, to an adult-onset disease with mild symptoms. Historically, based on the age of onset and the best motor function achieved, 5 types of SMA have been distinguished: SMA0, SMA1, SMA2, SMA3, and SMA4 [9]. The SMA 0 type is placed at the most severe end of the disease spectrum. These patients present with a prenatal onset, arthrogryposis and severe respiratory failure at birth. SMA1 is the most common type of SMA. In the natural course of the disease, children with SMA1 never achieve ability to sit independently and their life span is limited due to a respiratory failure. In SMA 2, patients can sit unsupported but are never able to walk. Patients with SMA3 achieve the ability to stand and walk independently, however the age of onset, severity of the disease as well as the age of immobilization varies substantially in this group. SMA 4 refers to patients with the onset usually after 30 years of age with a mild phenotype of disease. Each type can be divided into sub-types with more severe or milder forms reflecting the continuum in the spectrum of the disease. SMA1 includes very severe type SMA1a, less severe SMA1b and SMA1c with prolonged survival. SMA1c patients can reach adulthood in some cases without gastrostomy or invasive ventilation. Patients with SMA2 can be divided into SMA2a or milder form SMA2b. SMA 3a and 3b refers to the patients with onset before 3 years of age or over 3 years, respectively The course and clinical presentation of SMA1c and SMA2a as well as SAM2b and SMA3a overlap even those patients differed in achievement of main motor milestones. This observation is especially evident in later stage of disease [10].

Natural history studies demonstrated progressive disease course in all types of SMA [10–15]. Nusinersen is a splice-switching antisense oligonucleotide that promotes exon-7 inclusion into the *SMN2* gene transcript [16, 17], thus increasing the amount of functional SMN protein [18]. It is the first disease-modifying drug for all SMA types, which was approved for use by the U.S. Food & Drug Administration and European Medicines Agency in 2016 and 2017, respectively. Since then, it has been used worldwide, with about 11,000 patients treated up to mid-2022 [19]. In Poland, nusinersen treatment has been reimbursed since January 1, 2019, irrespective of patient age or SMA type and severity. Actually more than 850 patients are treated with nusinersen in Poland, another 120 receive other DMTs, accounting in total for about 80–85% of the whole population of Polish SMA patients. So far, the effectiveness and safety of nusinersen was demonstrated in clinical trials including pediatric patients only [18, 20] and several recent studies reported real-world data on the effects of nusinersen treatment in the adult population. Most studies indicated benefits of nusinersen in adults regardless of the disease type, duration, and severity. However, most of them reported outcomes for a follow-up duration of up to 14 months, while data on long-term nusinersen treatment are limited [19, 21–25].

The aim of this real-world study was to investigate the safety and effectiveness of nusinersen treatment in patients with a wide spectrum of SMA severity, followed for up to 30 months. Additionally, we aimed to assess the subjective opinion of patients on the effect of nusinersen treatment on their disease course and symptoms.

## Patients and methods

We prospectively assessed 130 patients who were treated with nusinersen between March 2019 and January 2022 when the data were cut. All patients received the treatment within the frame of a national reimbursement program at two centers that treat adults and children older than 5 years old.

The inclusion criteria were defined by the national reimbursement program of nusinersen treatment in Poland and were the following: patients presented clinically with SMA types (1c-3; classification based on the highest motor milestone achievement), diagnosis was confirmed by genetic testing, with assessment of the number of *SMN2* copies, the patients had no contraindication to lumbar punction or inability for lumbar punction. The program allows continuation of treatment in patients who started nusinersen before 2019, including Expanded Access Program (EAP). Additional criteria for inclusion into the study was the minimum and maximum treatment duration between 6 [T6] and 30 [T30] months, respectively. The patients were included into the treatment on a first-come, first-served basis from the region assigned to each center.

### Nusinersen administration

All patients were treated with intrathecal loading doses of 12-mg nusinersen at days 1 (T0, baseline), 14, 28, and 63, followed by maintenance doses every 4 months (from month 6 [T6] to month 30 [T30]) according to the standard protocol. Intrathecal drug administration was performed by an experienced neurologist using a conventional lumbar puncture (LP) or by radiologist using computed tomography (CT)–guided LP with an ultra-low dose of radiation (a procedure developed by our team and reported previously [26]) or the C-arm fluoroscopy system. Local anesthesia (5% lidocaine/prilocaine cream) or sedation was offered to all patients and used if needed. Patients were monitored for at least 5 h after each procedure for possible adverse events.

### Functional assessment

The Hammersmith Functional Motor Scale Expanded (HFMSE; score, 0–66), the Children’s Hospital of Philadelphia Infant Test of Neuromuscular disorders (CHOP-INTEND; score, 0–66), Revised Upper Limb Module (RULM; score 0–37), and the 6-minute walk test (6MWT) were used to evaluate patients depending on functional ability or disease severity [27–29]. In line with requirements of the national nusinersen reimbursement program, the HFMSE or CHOP-INTEND assessment was obligatory. RULM and 6MWT were additionally performed in one of the participating centers (Medical University of Warsaw, MUW) only, as there were not required by the national reimbursement program.

The patients who were able to walk or sit independently underwent assessment by HFMSE test. Those who presented with severe muscle weakness: never sit independently (SMA1) or who lost this ability in course of disease or were weak sitters (SMA2, SMA3) were assessed by CHOP-INTEND adapted to adult patients. The assessment by CHOP-INTEND test was approved and required in the national nusinersen treatment program. The RULM test was applied to patients who sit or walked independently.

Clinically significant improvement for the HFMSE, CHOP-INTEND, and RULM was defined as a change in the score of ≥ 3 points, ≥ 4 points, and ≥ 2 points, respectively [30–32]. For patients able to walk independently, significant improvement in the 6MWT was defined as an increase in walking distance by at least 30 m [29].

The assessments were performed by experienced physiotherapists at T0 and at administration of each maintenance dose from T6. Whenever possible, the patients were tested by the same physiotherapist. Data on adverse events, including headache, nausea, vomiting, vertigo, fever, back pain with assessment of duration and intensity were collected using a questionnaire at each point of treatment. Information on hospitalization due to adverse event was also collected. It was also possible to report other adverse event. The subjective assessment of treatment by patients (or caregivers in the case of children) was performed using the 7-point Patient Global Impression – Improvement (PGI-I) scale [33] rated as follows: very much improved (1); much improved (2); minimally improved (3); no change (4); minimally worse (5); much worse (6); and very much worse (7). Patients assessed their clinical status at each time point of nusinersen treatment versus baseline (T0).

### Ethics and patient consent

Patients or their caregivers, as appropriate, gave their informed consent for nusinersen treatment (National Health System form for reimbursed treatment program) and for data collection (Ethic Committee approval- BK/180/2008).

### Statistical analysis

The results of functional assessments were presented as mean, SD, and 95% confidence intervals (CIs), and percentage of patients who showed improvement after treatment. The statistical inference of differences was assessed using the Wilcoxon signed-rank test and a paired t-test. Multivariate linear regression (least squares estimation) was used to identify factors responsible for the differences versus baseline. The initial regressions analysis model included numerous factors, such as age at onset, duration of the disease to the first dose, age at first dose, initial scores on motor function scales, number of SMN2 copies, dummy variables indicating BMI score < 18.5 and > 25 for low/high body mass index. A general-to-specific modelling strategy was used to obtain the final regression model. Specifically, nonsignificant factors were removed from the model. Due to a relatively large sample size, Student-t test were used to verify the significance of associations between explanatory variables and the outcome, with a p value of less than 0.05 level considered significant. Statistical calculations were performed with Stata 14. In addition to a full-sample analysis, the results were also reported separately for: (1) specific SMA types (1c-3); (2) sitting SMA2 and ambulant and non-ambulant SMA3.

## Results

The final analysis included 120 treatment-naive patients. Of 130 screened patients, 7 were excluded due to an insufficient follow-up duration, and 3 patients were excluded due to treatment discontinuation, including a 12-year-old boy with SMA3 who entered a clinical trial, a 24-year-old woman with SMA3 who did not tolerate LP procedure, and a 26-year-old man with SMA1 who died before the fifth nusinersen dose due to tracheostomy bleeding unrelated to treatment. The first patient included in the analysis received the first nusinersen dose within the national reimbursement program on April 30, 2019, and the last patient started treatment on June 22, 2021. Most patients (n = 76.63%) have started treatment during the first 10 months since April 2019. 7 of 120 patients started the treatment earlier, in 2017–2018, within the frame of the EAP. All have SMA1c. Six of them (adults) started nusinersen treatment in Belgium then were transferred to continue EAP in Poland (MUW) staring in September 2018 and continue the treatment in National Health Service program. One SMA1c patient (teenager), started nusinersen treatment in EAP in Poland in one of the pediatric centers and then was transferred to MUW center. The information on their functional assessment at the beginning of treatment (T0) were available in the patients’ medical records. The mean treatment duration in the EAP those 7 patients was 11 months (range, 6–14 months) and involved an administration of 6 doses on average. All but one patient were treated in the reimbursement program for at least 600 days (about 20 months).

The number of assessed patients decreased over time because they did not reach a given time point before the data were cut. Additionally, due to covid pandemic restrictions some patients skipped the functional assessment at some points of treatment. The number of patients assessed at each time point by two main tests is shown in Additional file 1 (Study Flow Diagram).

The baseline characteristics of patients are presented in Table 1. Among the 120 patients included in the analysis, 53 were female and 67 were male. Most patients were adults (88%, 105 patients). The mean age at T0 was 32 years (SD, 14 years; range, 5–66 years). Among 15 children (1 SMA1c, 4 SMA2 and 10 SMA3) included in the study the mean age at T0 was 9.3 years (SD 3.6, median 8 years; range 5-17years). Eleven children were in age range 5–11 years and remining 5 children in the range 12–17 years. SMA1c was reported in 12 patients (10%); SMA2, in 19 (16%); SMA3, in 89 (74%). The SMA3 group was divided into sitters (41 patients) and walkers (48 patients). In the SMA1c group, 11 of the 12 patients were adults. Their mean age at T0 and a mean disease duration to the first dose was similar (because of the onset in the first months of life) and was 29 years (SD, 7.8 years; range, 13–45 years). The mean treatment duration for the whole study group was 23 months (SD, 14 months).

**Table 1:** Baseline characteristics of patients

Parameter			All patients n = 120 (100%)	SMA1c n = 12 (10%)	SMA2 n = 19 (16%)	SMA3 n = 89 (74%)
Sex, n (%)	Female		53 (44)	4 (33)	11 (58)	38 (43)
	Male		67 (56)	8 (67)	8 (42)	51 (57)
Age at onset, months, mean (median; min-max)			67 (75; 1-324)	3.7 (1.6; 1–7)	9.6 (3.5; 6–18)	82 (79; 1-324)
Age at baseline, years, mean (median; min-max)			32 (14; 5–66)	29 (28; 13–45)	24 (9.1; 5–41)	34 (14; 6–66)
Disease duration at baseline, years, mean (median; min-max)			27 (13; 3–61)	29 (28; 13–45)	23 (11; 4–41)	27 (14; 3–61)
*SMN2* copy number, n (%)		2	4 (3)	2 (17)	2 (11)	0
		3	71 (59)	9 (75)	16 (84)	46 (52)
		4	43 (36)	1 (8)	1(5)	41 (46)
		> 4	2 (2)	0	0	2 (2)
Ambulant, n (%)			NA	NA	NA	48 (54)
Age at loss of ambulation in 41 non-ambulant SMA3 patients, years, mean (median; min-max)			NA	NA	NA	19 (14; 1.5–61)
Scoliosis, n (%)			67 (56)	12 (100)	19 (100)	36 (40)
Scoliosis surgery, n (%)			13 (11)	0	6 (32)	7 (8)
NIV n (%)			17 (14)	10 (83)	4 (21)	3 (3)
IV n (%)			2 (2)	0	2 (10)	0
BMI, kg/m^2^ mean (SD; median; min-max)			22.1 (5.7; 22.2; 8.3–42.1)	16.3 (6; 16.0; 8.3–25.4)	20 (5.3; 19.5; 12.4–33)	23.3 (5.2; 23.1; 13.3–42)

BMI - body mass index; NA - not applicable; SMA - spinal muscular atrophy; NIV- non- invasive ventilation; IV- invasive ventilation

### Lumbar puncture procedures

A total of 1023 intrathecal drug administrations via LP were performed during the study. Conventional intrathecal administration was performed in 87 of 120 patients (77%) and included 746 LPs. Remining 277 intrathecal administration of nusinersen was performed using CT-guided LP (in 30 patients) or the C-arm X-ray system (in 3 patients) due to history of scoliosis surgery (12 patients), severe scoliosis (19 patients) and obesity (2 patients). These additional procedures for drug administration were required in, 75% (9 patients), 63% (12 patients and 14% (12 patients) with SMA1c, SMA2 and with SMA3, respectively. There were no administration failures. In 2 patients, LPs were performed via the intervertebral foramen using CT.

### Hammersmith functional motor scale expanded

The HFMSE assessment at T0 was performed in 73 patients (43 men), including 6 patients (4 children) with SMA2 and 67 patients (10 children) with SMA3 including 19 SMA3 non-ambulant patients (see Table 2). Their mean age and the mean disease duration at T0 was 31 years (SD, 15.6 years; range, 5–66 years) and 23.7 years (SD, 14 years; range, 4–62 years), respectively. One patient with SMA2 did not undergo assessment at day 180 (T6) but was assessed at the subsequent 4 time points. Therefore, he was included in the analysis. At T30, 28 patients were evaluated using the HFMSE.

**Table 2:** Baseline characteristic and demographics of analyzed patients at each time point of treatment: HFMSE assessment

	T6	T10	T14	T18	T22	T26	T30
Sex							
F (%)	29 (40)	25 (38)	25 (38)	25 (40)	20 (36)	18 (42)	10 (36)
M (%)	43 (60)	41 (62)	40 (62)	38 (60)	36 (64)	25 (58)	18 (64)
Age at treatment, years	31.2 (15.5; 5–67)	31.5 (15.6; 5–67)	31.8 (15.5; 6–67)	32.2 (15.5; 6–68)	32.5 (15.6; 6–68)	32.8 (15.5; 7–68)	33.2 (15.5; 7–69)
*SMN2* copy number							
2	1 (1)	2 (3)	2 (3)	2 (3)	2 (4)	1(2)	0
3	30 (42)	26 (39)	27 (42)	28 (44)	24 (42)	17 (40)	9 (32)
4	39 (54)	36 (55)	34 (52)	32 (51)	29 (52)	25 (58)	19 (68)
>4	2 (3)	2 (3)	2 (3)	1 (2)	1 (2)	0	0
SMA type							
1c	0	0	0	0	0	0	0
2	5 (7)	6 (9)	6 (9)	6 ( 10)	6 (11)	5 (12)	3 (11)
3	67 (93)	60 (91)	59 (91)	57 (90)	50 (89)	38 (88)	25 (89)
Ambulant	48 (67)	44 (67)	42 (65)	40 (63)	36 (64)	28 (65)	20 (71)
Baseline HFMSE score of 66	34.4 (17.9; 3–64)	33.9 (17.9; 3–64)	33.9 (18.5; 3–64)	33.3 (18.8; 3–64)	34.0 (18.8; 3–64)	35.3 (18.9; 3–64)	35.8 (18.1; 3–64)

Data are n (%), or Mean (SD, range). HFMSE -Hammersmith Functional Motor Scale Expanded

At least 1-point improvement was noted in 52 of the 72 patients (72%) at T6 versus T0, and in 24 of the 28 patients (86%) at T30 versus T0. Clinically meaningful improvement (≥ 3 points) in the HFMSE score was observed in 26 of the 72 patients (36%) after six months of treatment (T6). The percentage of responders gradually increased to 71% (20 of the 28 patients) at T30 versus T0 (Additional file 2).

In 11 of the 73 patients (15%), the HFMSE score improved by at least 10 points during the treatment. Of those patients, 8 were still able to walk, 6 had 4 copies of *SMN2*, and 5 had 3 copies of *SMN2*.

Of the 73 patients, 4 had a score of ≥ 60 points at T0. Two patients who scored 64 points at T0 remained stable up to T26 and T30, respectively. One patient improved from 60 to 63 points at T14 and was stable until T22, and 1 patient improved from 61 to 63 points at T14 and was stable at T30.

Worsening was observed in 8% of patients at T6 and 4% of patients at T30 (Additional file 2). Similar results were obtained in a separate analysis for the SMA3 group (Additional file 3). The separate, statistical analysis for SMA2 was not performed because of a small number of those patients (n = 6). Three of SMA2 patients were assessed until T30; 2 of them improved (one from 20 to 29 points, and the second from 17 to 21 points), and the third was stable (8 points at T0 and at T30). The other 2 SMA2 patients were assessed until T26 and both improved (one from 9 to 11, the other from 4 to 6 points). The sixth patient, the only one in whom worsening was observed, was treated until T22 and his score was 4 at T0 and 3 at T22.

A mean HFMSE score for 73 patients at T0 was 34.0 points and gradually increased at subsequent time points of nusinersen treatment up to 40.9 points at T30 The mean value of differences between T0 and T6 was 2.5 points and doubled to 5.1 points at T30. The mean differences between T0 and each time point of treatment reached the statistic significances (p < 0.001).

**Fig. 1:**
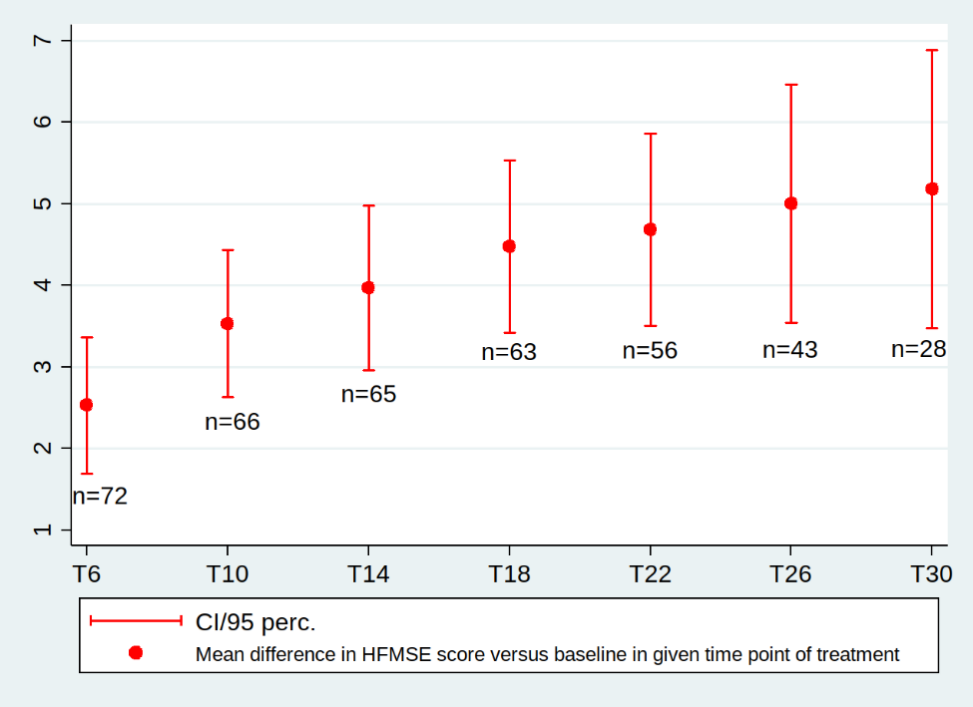
Mean differences in HFMSE score between the baseline (T0) and subsequent treatment time points (in months) up to T30, p < 0.001 at all time points; n- number of patients assessed at each time point of treatment

The results are presented in Table 3; Fig. 1 [see also Additional file 4] .

**Table 3:** Changes in the HFMSE score versus baseline (6 patients with SMA2, 67 patients with SMA3 included 48 ambulant and 19 non-ambulant patients)

Changes in HFMSE v T0	Month of treatment (no. of patients)							
	T0 (73)	T6 (72)	T10 (66)	T14 (65)	T18 (63)	T22 (56)	T26 (43)	T30 (28)
Mean score (median, SD, min-max)	34.0 (36, 18, 23–64)	37.0 (36, 17.7, 3–64)	37.5 (39, 17.7, 4–64)	37.9 (40, 18.4, 3–64)	37.8 (39, 18.6, 3–64)	38.7 (41.5, 18.6, 3–64)	40.3 (42, 18.1, 5–64)	40.9 (42, 16.5, 5–64)
Mean differences vs. baseline (median, SD, min-max)	NA	2.5 **(**2, 3.6; -6-19)	3.5 (3, 3.7; -1-19)	3.9 (3, 4.1; -4-19)	4.5 (4, 4.3; -1-20)	4.7 (4, 4.5; -4-20)	5.0 (4, 4.8; -1-20)	5.1 (5, 4.6; -1-18)
Mean differences vs. baseline (95% CI)	NA	2.5 (95%; 1.66–3.39)	3.5 (95% 2.59–4.46)	3.9 (95%; 2.92–5.01)	4.5 (95%; 3.3–5.5)	4.7 (95%; 3.4–5.9)	5.0 (95%; 3.5–6.5)	5.1 (95%; 3.4–6.9)
p value*	NA	< 0.001	< 0.001	< 0.001	< 0.001	< 0.001	< 0.001	< 0.001

*p value was assessed by the Wilcoxon and Student-t tests, and the results were the same

NA – not applicable

Additionally, there were significant differences in mean HFMSE scores between subsequent time points during the follow-up, starting from T6, with a continuous increase up to T30 (Additional file 5) .

The mean HFMSE score changes between baseline and each time point of treatment assessed separately for ambulant (48) and non-ambulant (25) patients revealed the statistically significant difference at each point of treatment for each group. However, when these results were compared, the statistically significant differences between ambulant and not-ambulant patients was not found in any point of treatment (p > 0.2) (Additional file 6).

### Children’s hospital of philadelphia infant test of neuromuscular disorders

Among 47 patients (24 men [51%]) assessed with CHOP-INTEND, 12 patients had SMA1; 13, SMA2; and 22, SMA3 (see Table 4). The mean age at T0 was 33.7 years (SD, 11.0; range, 13–66), the mean disease duration to the first dose was 31.7 years (SD, 9.5; range, 3.0–58.0). Forty-four patients were assessed at least at T0 and T6. The baseline CHOP-INTEND score was not available for the 3 adults with SMA1 who started treatment abroad within the EAP. They started evaluation in the study at T10, T14, and T18, respectively. In two of them the assessment was available up to T30. The data are presented in a separate analysis of SMA1 patients (Additional file 7). In the patients assessed by CHOP-INTEND an improvement by at least 1 point was noted in 77% (34 of 44) of patients at T6 and in 94% (16 of 17) of patients at T26 vs. baseline The clinically meaningful improvements (≥ 4 points) in the CHOP-INTEND score was observed in 20.5% (9 of 44) at T6 and in 65% (11 of 17) at T26 (Additional file 8).

At T30, only 5 patients were assessed and improvement versus baseline was noted in 4 (all SMA1). In separate analyses for SMA1, SMA2 and SMA3patients, the highest percentage of patients who improved at each time point of treatment was noted for SMA3 (Additional files 7, 9, 10).

**Table 4:** Baseline characteristic and demographics of analyzed patients at each time point of treatment: CHOP-INTEND assessment

	T6	T10	T14	T18	T22	T26	T30
Sex							
F (%)	22 (50)	21 (51)	18 (47)	18 (49)	11 (42)	6 (35)	3(60)
M (%)	22 (50)	20 (49)	20 (53)	19 (51)	15 (58)	11 (65)	2(40)
Age at treatment, years	33.6 (11.2; 14–66)	33.9 (11.1; 14–66)	34.3 (11.2; 14–67)	34.6 (11.1; 15–67)	34.9 (11.1; 15–67)	35.3 (11.2; 15–68)	35.6 (11.1; 16–67)
*SMN2* copy number							
2	0	0	0	0	0	0	0
3	40 (91)	37 (90)	34 (89)	33 (89)	24 (92)	16 (94)	5 (100)
4	4 (9)	4 (10)	4 (11)	4 (11)	2 (8)	1 (6)	0
>4	0	0	0	0	0	0	0
SMA type							
1c	9 (20)	9 (22)	8 (21)	8 (22)	8 (31)	8 (47)	4 (80)
2	13 (30)	11 (27)	11 (29)	11 (30)	8 (31)	5 (29)	1 (20)
3	22 (50)	21 (51)	19 (50)	18 (49)	10 (38)	4 (24)	0
Baseline HFMSE score of 66	34.4 (17.9; 3–64)	33.9 (17.9; 3–64)	33.9 (18.5; 3–64)	33.3 (18.8; 3–64)	34.0 (18.8; 3–64)	35.3 (18.9; 3–64)	35.8 (18.1; 3–64)

Data are n (%), or Mean (SD, range). CHOP-INTEND-Children’s Hospital of Philadelphia Infant Test of Neuromuscular Disorders

The mean CHOP-INTEND score increased significantly between T0 (24.2 points) and subsequent time points up to T26 (28.3 points). At T30, only 5 patients were available for assessment, and the mean difference was 9.4 points (95%CI; 0.17–18.63; P = 0.12) (Table 5).

**Fig. 2:**
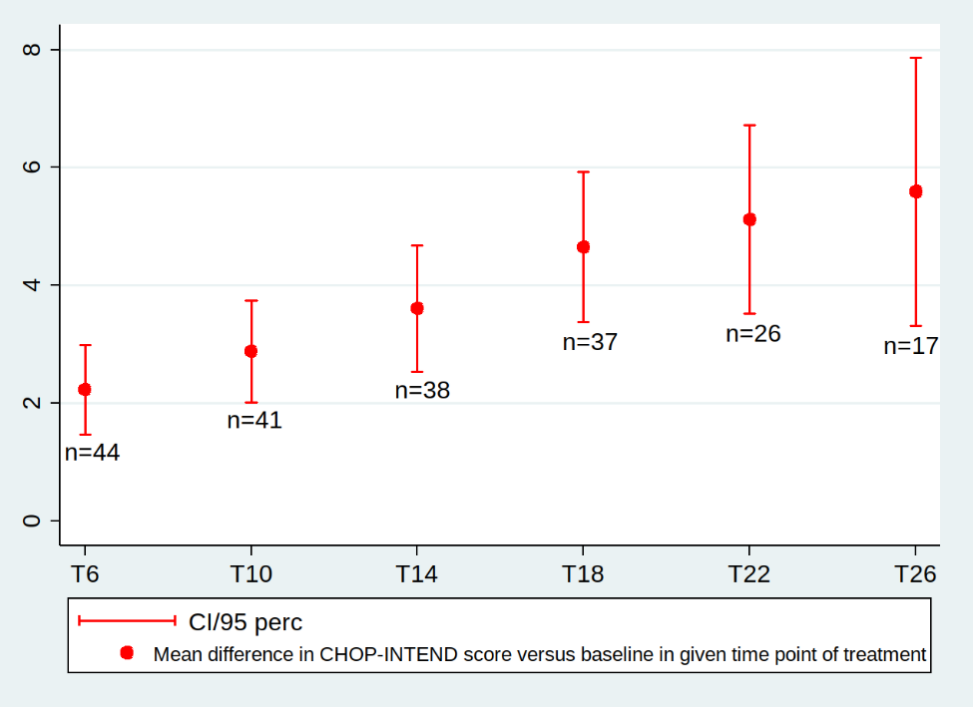
Mean differences in CHOP-INTEND score between the baseline (T0) and subsequent treatment time points (in months) up to T26, p < 0.001 at all time points; n- number of patients assessed at each time point of treatment

The mean value of differences between T0 and T6 was 2.23 points and increased to 5.59 points at T26. The mean differences between T0 and each time point of treatment up to T30 reached the statistic significances (p < 0.001) (Table 5; Fig. 2).

**Table 5:** Changes in the CHOP-INTEND score (max. 64 points) versus baseline (T0)

Changes in CHOP-INTEND v T0	Month of treatment (no. of patients)							
	T0 (44)	T6 (44)	T10 (41)	T14 (38)	T18 (37)	T22 (26)	T26 (17)	T30 (5)
Mean score (median, SD, min-max)	24.2 (23.5, 9.5, 3–49,)	26.4 (27.5, 9.8, 6–49)	27.2 (30, 9.8, 9–51)	27.8 (28.5, 9.5, 12–51)	28.6 (39, 9.6, 12–51)	28.3 (30, 10.5, 12–51)	28.3 (27, 10.5, 12–51,)	28.3 (25, 5.7, 20–31)
Mean differences vs. baseline (median, SD, min-max)	NA	2.23 (2, 2.56, -1-11)	2.88 (3.5, 2.84, -1-14)	3.61 (5, 3.37, 0–17)	4.65 (3.5, 4.0, 0–17)	5.11 (5, 4.16, 0–18)	5.59 (5, 4.78, -2-17)	9.4 (9, 7.44, -1-17)
Mean differences vs. baseline (95% CI)	NA	2.23 (1.45–3.01)	2.88 (1.98–3.77)	3.61 (2.5–4.71)	4.65 (3.33–5.97)	5.11 (3.44–6.80)	5.59 (3.13–8.05)	9.4 (0.17–18.63
p value*	NA	< 0.001	< 0.001	< 0.001	< 0.001	< 0.001	< 0.001	0.12 and 0.024 (only 5 patients)*

*p value was assessed by the Wilcoxon and Student-t tests, and the results were the same except for T30 – Wilcoxon test, p = 0.12; Student-t test, p = 0.024 (only 5 patients) NA-not applicable

There were statistically significant differences in the mean CHOP-INTEND score between subsequent time points from T6 up to T22. Between T26-T30, the score increased by 0.8 (p = 0.25), but only 5 patients were assessed (Additional file 5).

**Fig. 3:**
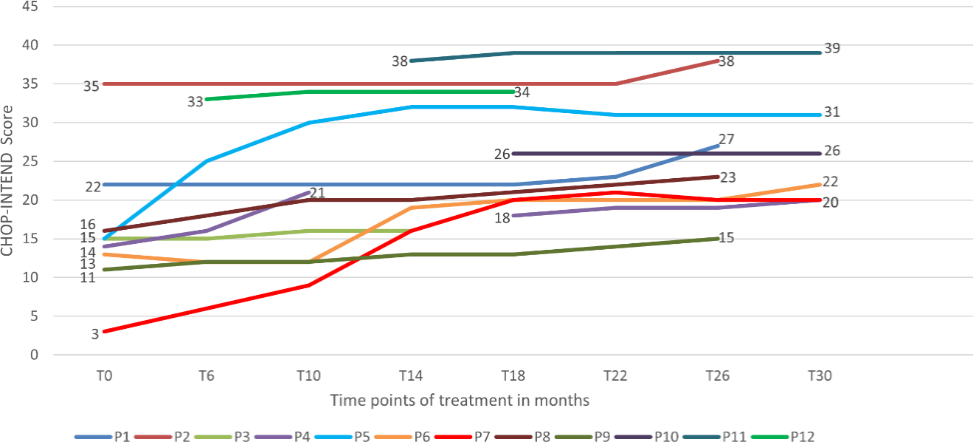
CHOP-INTEND results in 12 patients with SMA1c (all except 1 [P4] were adult at T0)

Of the 12 patients with SMA1, 9 were assessed at T0 and at were treated at least ten months (assessment at T10), and all of them showed improvement in the CHOP-INTEND score by at least 1 point (Fig. 3). Eight of these patients were assessed at T26, and clinically meaningful improvement (≥ 4 points) was shown in 58.3% (range, 5–17 points). All 4 patients who were assessed at T0 and at T30 showed improvement by more than 4 points (range, 6–17 points). Of the 3 patients without assessment at T0, 2 patients showed improvement by 1 point, and 1 patient was stable during the follow-up (Fig. 3).

### Revised upper limb module

Fifty-one patients (9 with SMA2 and 43 with SMA 3; 30 men [59%]) were assessed by the RULM at T0 and at least at one time point of treatment from T6 to T30. The mean age at T0 was 27 years (median, 30; SD, 14; range, 5–66), and a mean disease duration to the first dose was 22 years (median, 24.5; SD, 12.8; range, 3.8–62.0). 25 patients were non-ambulant (9 with SMA 2 and 16 with SMA3) and 26 were ambulant.

Of the 51 patients, 11 (21.5%) had the maximum score (37 points) at T0, and it was maintained during the follow-up. In 6 of those11 patients, the last evaluation was at T30; in 2 patients, at T26; and in the remaining 3 patients, at earlier time points. Additionally, 8 patients reached the maximum score during treatment, but their score at baseline was at least 34 points.

At T6 and T10, 20 of the 51 patients were assessed. Improvement was observed in 30% at both time points. During further treatment, the percentage of patients who improved increased to 50% (17 of the 34 patients) at T14, 53.5% (23 of 43) at T18, 57% (21 of 37) at T22, 59% (20 of 34) at T26, and 61% (14 of 23) at T30. The number and percentage of patients who achieved clinically meaningful improvement (≥ 2 points) in RULM was 26% (5 of 20) at T6 and increased to 43.5% (10 of 23) (Additional file 11).

The mean RULM score significantly increased between T0 and subsequent time points up to T30, except between T0 and T10 (Table 6). Similar results were obtained when the patients with the maximum score at baseline were excluded. Differences in the mean RULM score between individual time points are shown in Additional file 5.

**Table 6:** Changes in the RULM score (max. 37 points) versus baseline (T0) NA- not applicable

	Months of treatment (no. of patients)							
	T0 (51)	T6 (20)	T10 (20)	T14 (34)	T18 (43)	T22 (37)	T26 (34)	T30 (23)
Mean score (median, SD, min-max)	26.5 (33, 11, 3–37)	23.3 (24.5, 13.2, 3–37)	26.0 (32.5, 12.5, 4–37,)	27.3 (30.5, 10.6, 3–37)	28.3 (32, 9.4, 5–37)	28.5 (31, 9.4, 4–37)	29.8 (32, 8.1, 11–37)	30.6 (33, 7.7, 12–37)
Differences in mean vs. baseline (median, SD, min-max)	NA	0.6 (0, 1.14 0.25, -1–3)	0.3 (0, 1.21 0.27, -2–4)	0.79 (0.5, 1.65 0.28, -4–6)	0.91 (1, 1.89 0.29, -2–6)	1.1 (1, 2.04 0.34, -2–6)	1.32 (1, 2.06, -3–7)	1.96 (1, 2.4, -1–8)
Differences in mean vs. baseline mean (95% CI)	NA	0.6 (0.1–1.1)	0.3 (0.3–0.9)	0.79 (0.2–1.4)	0.9 (0.3–1.5)	1.1 (0.4–1.8)	1.32 (0.6-2.0)	1.96 (0.9-3)
p value*	NA	0.047	0.37	0.001	0.003	0.001	< 0.001	< 0.001

*Wilcoxon test; NA- not applicable

The mean RULM score changes between baseline and each time point of treatment assessed separately for ambulant (26) and non-ambulant (25) patients revealed the statistically significant difference at each point of treatment for ambulant patients. In non-ambulant patients the significant improvement is observed only after T22. The differences in mean score between ambulant and not-ambulant patients was statistically significant in the period T14-T26 and it is very closed to statistical significant at T30. The data showed that non-ambulant patients gained better improvement (Additional file 12).

### 6-minute walk test

Twenty-seven patients with SMA3 (18 men [67%]) were evaluated by the 6MWT at T0 and at least 1 time point of treatment from T6 to T30. The lack of a fairly significant number of ratings in the 6MWT test was mainly due to patients’ fear of staying too long in the hospital and contacting medical staff and other patients during the pandemic. The mean age of these patients at T0 was 27 years (SD, 13; range, 6–59), and the mean disease duration was 18 years (SD, 10; range, 4–33).

Clinically meaningful improvement (change in 6MWT ≥ 30 m) was observed in 33% (5 of 15) at T6, and these values gradually increased to 50% (6 of 12) at T30. The number and percentage of patients with any worsening was relatively large in each point of treatment. At T6 was 40% (6 of 15) and at T30 was 33% (4 of 12) (Additional file 13).

Statistically significant improvement in 6MWT results was observed for T10 (22.1, p = 0.007), T14 (16.6, p = 0.041), and T18 (18.1, p = 0.028) versus T0. At other time points, no significant differences were found (Table 7). No significant differences were observed between subsequent time points of treatment (Additional file 5).

**Table 7:** Changes in 6MWT results versus baseline. NA- not applicable

	Month of treatment (no. of patients)							
	T0 (27)	T6 (15)	T10 (14)	T14 (19)	T18 (22)	T22 (18)	T26 (16)	T30 (12)
Mean score (median, min-max, SD)	267.9 (265.5, 25–593.5, 157.7)	307.8 (281, 30–548, 157.1)	312.3 (314, 100–548, 104.7)	296.9 (308, 30–618, 173)	279.5 (292.5, 39–639, 174.1)	287.2 (264.5, 53–625, 168.7)	289.7 (288, 24–612, 184.9)	245.7 (223.5, 40–575, 159.8)
Mean differences vs. baseline (median, SD, min-max)	NA	5.4 (12, 36.4, -61–63.5)	22.1, (21, 26.8–13.5–91.5)	16.6 (17, 32, -37.5–96.5)	18.1 (22, 43.3, -95–98.5)	9.9 (-0.5, 50.5, -121-100.5)	24.0 (20.8, 61.0, -139–105.5)	27.0 (33, 67.1, -111.5–120.5)
Mean differences vs. baseline (95% CI)	NA	5.4(-14.8–3.01)	22.1(6.6–37.6)	16.6(1.2–32.1)	18.1(-1.0–37.3)	9.9(-15.2–35.0)	24.0(-8.5–56.6)	27.0(-15.7–69.6)
p value	NA	p = 0.288	p = 0.007	p = 0.041	p = 0.028	0.449	0.078	0.204

### Multivariate regression

Multivariate regression analyses with changes in the HFMSE score as an outcome variable showed that improvement in the first period of treatment (T0-T6, T0-T10) depended on sex, with women showing a greater improvement (p = 0.038, p = 0.010, respectively). The improvement in the longer horizon (T0-T26, T0-T30) is negatively associated with initial score on motor scale (p = 0.046, p = 0.018). None of the additional factors (number of the SMN2 copies, age at onset, duration of the disease to the first dose and age at the first dose, body mass index) showed a significant correlation with the treatment outcome (Additional file 14).

Multivariate regression analyses with changes in the CHOP-INTEND score as an outcome variable did not show any significant association with factors tested in the HFMSE (data not shown).

### Safety

Data on adverse events after LP and drug administration also included the loading doses (days 1, 15, 30, and 63) and were available for 1023 intrathecal injections. The procedure was generally well tolerated. Post lumbar puncture syndrome (PLPS) was observed in 198 of 1023 (19%) LPs. All patients with PLPS reported headache, mainly of mild intensity. Back pain was reported for 111 LP procedures (11%). Nausea was reported by 41 patients (4%) and vomiting by 12 (1%). Only 1 patient (SMA1) required a single hospitalization for severe back pain after LP. PLPS developed on the same day, on the second day, or on the third day after LP in 13%, 67%, and 13% of all LPs, respectively. In 7% of LPs, PLPS occurred after 3 or more days after the procedure but not later than after 7 days.

The LP procedure supported by CT or the C-arm fluoroscopy system was associated with a lower risk of PLPS compared with conventional intrathecal drug administration (11% vs. 22%, respectively; p < 0.00001).

In one case (a 26-year-old woman with SMA2 and history of scoliosis surgery), cerebrospinal fluid leak was observed after CT-guided injection at T10. The leak stopped within 1 h without intervention.

### Patient global impression – improvement

Overall, 96.5–100% of patients reported subjective improvement or stabilization. During the 30 months of treatment, none of the patients reported feeling much or very much worse (grades 6 or 7) (Additional file 15).

The distribution of responders, that is, patients who achieved a clinically meaningful improvement in each of the functional tests, is shown in Table 8.

**Table 8:** Distribution of patients who achieved clinically meaningful improvement (responders) in each of the functional tests applied in the study. For the PGI-I, responders were defined as patients who improved minimally, much, or very much

Functional test	Total no. of patients no. of responders, % of responders	Month of treatment						
		T6	T10	T14	T18	T22	T26	T30
HFMSE, n = 73 (SMA2, 6; SMA3, 67)	Total	72	66	65	63	56	43	28
	Responders	26	35	40	38	35	26	20
	% of responders	**36**	**53**	**62**	**60**	**63**	**60**	**71**
CHOP-INTEND, n = 44* (SMA1c, 9; SMA2, 13; SMA3, 22)	Total	44	41	38	37	26	17	5
	Responders	9	15	19	23	16	11	4
	% of responders	**20.5**	**37**	**50**	**62**	**62**	**65**	**80**
RULM, n = 51 (SMA2, 9; SMA3, 43)	Total	20	20	34	43	37	34	23
	Responders	5	2	9	11	8	13	10
	% of responders	**25**	**10**	**26.6**	**25.5**	**22**	**38**	**43.5**
6MWT, n = 27 (all SMA3)	Total	15	14	19	22	18	16	12
	Responders	5	4	5	7	7	7	6
	% of responders	**33**	**29**	**26**	**32**	**39**	**44**	**50**
PGI-I, n = 120	Total	120	116	110	104	89	64	47
	Responders	95	90	82	78	68	56	40
	% of responders	**79**	**78**	**76**	**75**	**76**	**87.5**	**85**

*Three patients who did not undergo assessment at T0 were excluded

Percentage of responders (R) at each time point of treatment for the HFMSE (≥ 3 points), CHOP-INTEND (≥ 4 points), RULM (≥ 2 points), 6MWT (≥ 30 m), and PGI-I (any subjective improvement).

## Discussion

Adults constitute about half of all patients with SMA [34]. Recently, numerous real-world studies reported the effectiveness and safety of nusinersen treatment in adults and older children [19, 22–24, 35, 36]. However, while the studies confirmed the beneficial effect and a satisfactory safety profile, the longest follow-up was limited to 14 months, and thus data on long-term effects in adults are limited. In addition, there was no evidence on the effectiveness of nusinersen in patients with SMA1 with prolonged survival up to adulthood, that is in SMA1c. The present study was performed with the aim to fill the gap in the current scientific knowledge. SMA1c adult patients are rarely viewed as eligible for treatment, as no data was reported so far in this patient’s group. SMA1c is a significantly a milder phenotype then SMA1a and SMA1b and the course and clinical presentation is similar to SMA2a phenotype especially in later stages of diseases [10].

Our study confirmed a significant improvement in mean HFMSE scores at 14 months versus baseline and demonstrated continued functional gain also after subsequent 16 months (T30) of nusinersen administration. Previous studies showed the beneficial effect of treatment at 14 months [21, 22]. Only few studies reported a longer observation time, but did not exceed 24 months [24, 25]. Our study showed significant differences in the mean HFMSE score between baseline and subsequent time points of treatment in all 73 patients, including 6 with SMA2 and 67 with SMA3. When patients with SMA3, ambulant SMA3, and non-ambulant SMA3 were evaluated separately, the differences in the mean score between baseline and subsequent time points were almost identical for all these groups. Interestingly, a recent study of 111 children and young adults with SMA2 and SMA3 (median age, 12.5 years) followed for 24 months showed different results [24]. There was a significant increase between baseline and 12 months in SMA2, but not in SMA3. Moreover, a significant increase was noted in HFMSE between baseline and 24 months in SMA2 and SMA3 only in children younger than 5 years (p = 0.009 and p = 0.043, respectively), but not in older subgroups. Our results demonstrated a significant potential for improvement also in older patients with SMA2 and SMA3, which stands in contrast to the natural history of SMA2 and SMA3, with a functional decline manifesting as a mean loss of 0.5 to 1 points in the HFMSE score per year [10–12].

Interestingly, although the most dynamic improvement in our study was observed during the first 18 months of treatment, it remained significant until the end of follow-up. The rate of responders as assessed by the HFMSE score increased to 71% (20 out of 28 patients) at T30. In an Italian study, the percentage of responders increased from 28% (33 of 116 patients) at T6 to 49% (25 of 51 patients) at T14 [21], while in a German cohort, it was only 40% (23 of 57) at T14 [22]. These differences may be due to a higher proportion of patients with SMA2 and a lower HFMSE score at baseline in those studies as compared with our cohort. High HFMSE scores at baseline predict better improvement, at least during the first 14 months of treatment [22]. The floor effect of HFMSE in weak sitters may affect the sensitivity to detect changes in adult patients and should be remember when interpreting the treatment results [25].

Our data support previous findings that even adult patients with poor motor function at baseline can derive significant benefits from nusinersen treatment [21, 23, 36]. We demonstrated improvement in patients with SMA1c and severe SMA2 and SMA3 who were assessed by the CHOP-INTEND test. A mean CHOP-INTEND score significantly increased between baseline and subsequent time points up to T26, with 80% of responders at T30. At T26, 7 of 8 patients with SMA1c achieved a clinically meaningful response. Moreover, all 4 SMA1c patients who reached T30 were responders. There are no literature data on nusinersen effectiveness in adult patients with SMA1c.

Upper limb function assessed with the RULM showed continuous improvement, not only during the first 14 months of treatment [21, 22], but also until T30. Again, our study demonstrated a greater benefit than previous reports [21, 24]. The percentage of responders increased from 25% (5 of 20 patients) at T6 to 43% (10 of 23 patients) at T30. All patients with a maximum score at baseline maintained their function. The ceiling effect of the score makes it difficult to demonstrate improvement by means of the RULM in patients with milder form of SMA [21, 24, 25, 36].

As for the 6MWT, our study indicated a continued benefit of treatment with stabilization after 18 months.

The multivariate regression analysis showed that during the first 10 months of treatment, women showed greater improvement in the HFSME score than men; however, this difference was not observed in the long-term follow-up. The improvement in the longer horizon (T0-T26, T0-T30) is negatively associated with initial score on motor scale (p = 0.046, p = 0.018), which is in line with previous studies [22]. The results concerning the association between changes in the HFMSE score and factors such as sex and initial HFMSE scale remain robust across regressions utilizing various sets of explanatory variables (with 0.1 < p < 0.01). It is important to note, however, that these findings should be interpreted with caution due to relatively small sample and the p-values within a range that indicates marginal statistical significance. None of the other factors/variables which were taken to account in the multivariate regression analyses did show a significant correlation with the treatment outcome. This observation was found also in previous research [21].

Our study confirmed that nusinersen administration is safe and well tolerated by patients, adverse events were seen in 30% of the patients but were mostly mild.This supports previous reports [21, 22, 35]. We observed that although a CT-guided LP requires a more complex medical approach, the risk of PLPS was significantly reduced in comparison with conventional LP. It could be related to LP technique (less traumatic, guided approach) but also the functional status of the patients, as guided technique was employed in more advance, non-ambulant patients.

The results of the PGI-I questionnaire confirmed a high level of patient satisfaction with treatment results [37].

Our study has several limitations. First, the size of adult SMA1c and SMA2 samples was relatively small. The CHOP-ATTEND test validated nowadays for adult patients with severe symptoms was not available at the time of the study. For this reason we applied in these cases CHOP-INTEND test which is not validated in the adults. However this was the only scale at that time available and recommended for use in non-sitters or very weak sitters, including adults [38, 39]. Additionally, the results of some functional tests were not available for all time points, due to restrictions imposed during the COVID19 pandemic in 2020 and 2021. During the pandemic 31 doses were delayed, and we were not able to control our analysis for this factor. Additionally, the study did not involve a control group of untreated patients as the national program of nusinersen treatment in Poland does not have significant exclusion criteria and most (currently over 900) patients with SMA are treated.

In conclusion, our data provide real-world evidence for continuous effectiveness and safety of long-term nusinersen treatment in adults and older children regardless of the type and severity of SMA, including adult patients with SMA1c.

### Electronic supplementary material

Below is the link to the electronic supplementary material.

Additional file 1: Study flow diagram for HFMSE and CHOP-INTEND tests.

Additional file 2: Changes versus baseline (T0) in all patients (n = 73) who were assessed by the Hammersmith Functional Rating Scale Expanded (HFMSE), including 6 patients with SMA2 and 67 patients with SMA3.

Additional file 3: Changes versus baseline (T0) in SMA3 patients (n = 67) who were assessed by the Hammersmith Functional Rating Scale Expanded (HFMSE), including 48 ambulant and 19 non-ambulant patients.

Additional file 4: Changes in HFMSE score from T0 (baseline) to T6 (6 months)[A], T10 (10 months)[B], T14 (14 months) [C], T18 (18 months) [D], T22 (22 months) [E], T26 (26 months) [F], T30 (30 months) [G]. Each bar represents a single patient. HFMSE = Hammersmith Functional Motor Scale.

Additional file 5: Differences in mean scores between subsequent treatment time points in all functional tests: HFMSE, CHOP-INTEND, RULM, 6MWT.

Additional file 6: Changes in the HFMSE score versus baseline (T0) for ambulant and non-ambulant patients and comparison the results.

Additional file 7: Changes versus baseline (T0) in SMA1 patients (n = 9) who were assessed by the CHOP-INTEND N -number of patients; (%) percentage of patients. 

Additional file 8: Changes versus baseline (T0) in all patients (n = 44) who were assessed by the Children’s Hospital of Philadelphia Infant Test of Neuromuscular Disorders (CHOP-INTEND).SMA1c (n = 9; additional 3 patients started evaluation in the study at T10, T14, and T18, respectively), SMA2 (n = 13), SMA3 (n = 22) patients.

Additional file 9: Changes versus baseline (T0) in SMA2 patients (n = 13) who were assessed by the CHOP-INTEND.

Additional file 10: Changes versus baseline (T0) in SMA3 patients (n = 22) who were assessed by CHOP-INTEND.

Additional file 11 Changes versus baseline (T0) in 51 patients (9 with SMA2 and 43 with SMA3) assessed by Revised Upper Limb Module (RULM); n = number of patients; (%) percentage of patients.

Additional file 12: Changes in the RULM score versus baseline for ambulant and non-ambulant patients and comparison the results.

Additional file 13: Changes in 6 min walk test (6MWT) results versus baseline (T0).

Additional file 14: Results of the multivariate regression (dependent variable: HFMSE score versus baseline-T0).

Additional file 15: Results for **Patient Global Impression – Improvement (PGI-I)** assessment at subsequent time points of treatment: data for 120 all patients.

## Data Availability

The datasets used and/or analyzed in the current study are available from the corresponding author on reasonable request.

## Declarations

**Ethics approval and consent to participate**.

Patients or their caregivers, as appropriate, gave their informed consent for nusinersen treatment (National Health System form for reimbursed treatment program) and for data collection (Ethic Committee approval- BK/180/2008).

**Consent for publication**.

### Authors’ information

The Department of Neurology at the Medical University of Warsaw, Poland, is a member of the European Reference Network for Rare Neuromuscular Diseases (EURO-NMD).

## Abbreviations

6MWT: 6-minute walk test
PGI-I: Patient Global Impression – Improvement
CHOP-INTEND: Children’s Hospital of Philadelphia Infant Test of Neuromuscular Disorders
CI: Confidence interval
HFMSE: Hammersmith Functional Motor Scale Expanded
LP: Lumbar puncture
RULM: Revised Upper Limb Module
SMA: Spinal muscular atrophy
