# Supplementary figures and images for: Long-term nusinersen treatment across a wide spectrum of spinal muscular atrophy severity: a real-world experience
Source: Orphanet J Rare Dis. 2023 Aug 4;18:230. doi: 10.1186/s13023-023-02769-4 (PMC10401775; doi:10.1186/s13023-023-02769-4)

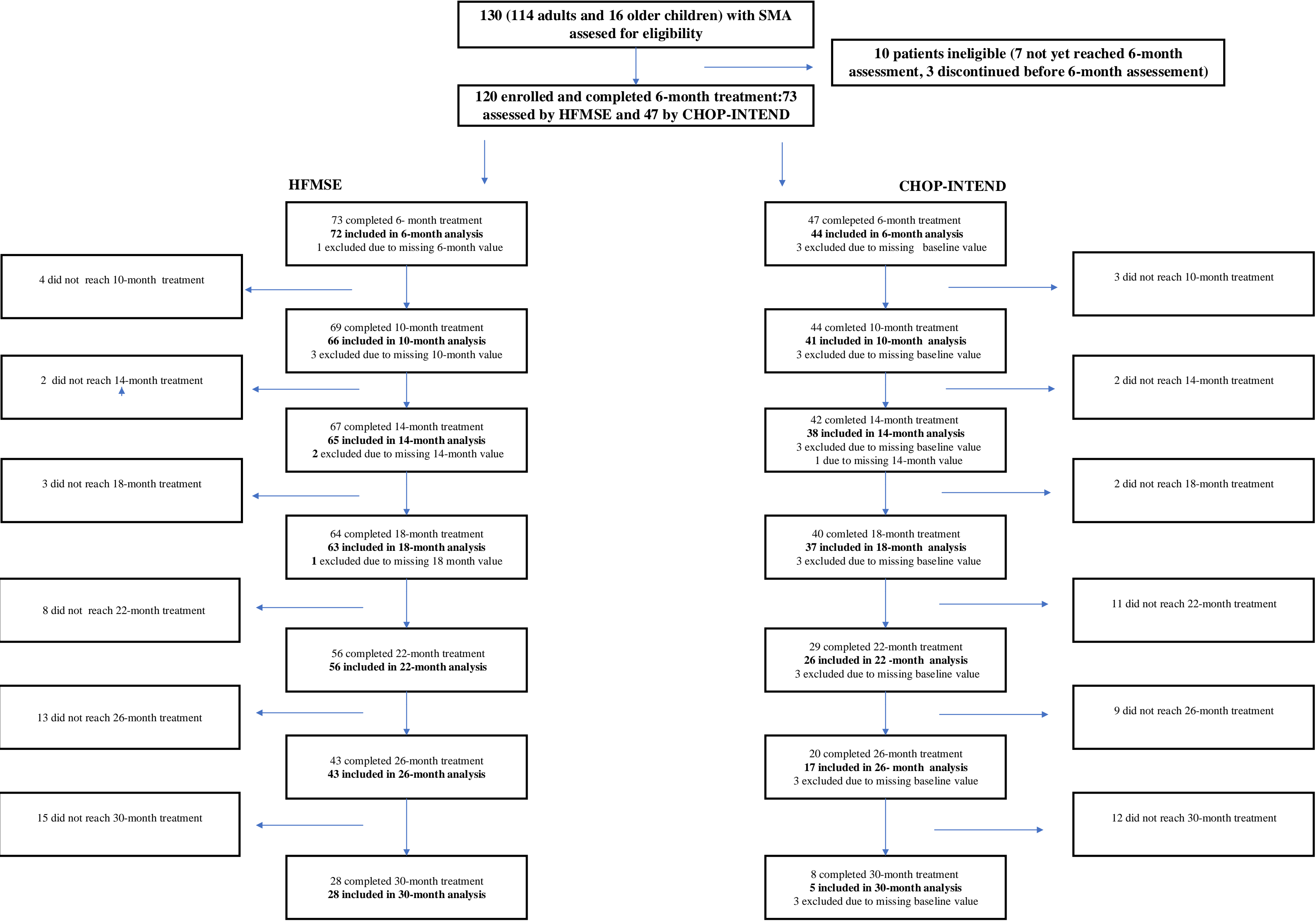

Additional file 1. Study flow diagram for HFMSE and CHOP-INTEND tests.

Supplement: Supplementary file 1 — Additional file 1: Study flow diagram for HFMSE and CHOP-INTEND tests. [file 13023_2023_2769_MOESM1_ESM.pdf]
