# Supplementary material for: Long-term nusinersen treatment across a wide spectrum of spinal muscular atrophy severity: a real-world experience
Source: Orphanet J Rare Dis. 2023 Aug 4;18:230. doi: 10.1186/s13023-023-02769-4 (PMC10401775; doi:10.1186/s13023-023-02769-4)
Supplement: Supplementary file 2 — Additional file 2: Changes versus baseline (T0) in all patients (n = 73) who were assessed by the Hammersmith Functional Rating Scale Expanded (HFMSE), including 6 patients with SMA2 and 67 patients with SMA3. [file 13023_2023_2769_MOESM2_ESM.docx]

**Additional file 2.** Changes versus baseline (T0) in all patients (n=73) who were assessed by the Hammersmith Functional Rating Scale Expanded (HFMSE), including 6 patients with SMA2 and 67 patients with SMA3.

| **Changes vs T0 in HFMSE for all 73 patients** | **Month of treatment (no. of patients)** | | | | | | |
| --- | --- | --- | --- | --- | --- | --- | --- |
|  | **T6**  **(72)*** | **T10 (66)** | **T14 (65)** | **T18 (63)** | **T22 (56)** | **T26 (43)** | **T30 (28)** |
| Worsening (change in HFMSE <0), n (%) | 6 (8) | 1 (1.5) | 4 (6) | 4 (6) | 3 (5) | 2 (5) | 1 (4) |
| Stable (change in HFMSE = 0), n (%) | 14 (19) | 13 (20) | 9 (14) | 5 (8) | 6 (11) | 5 (12) | 3 (11) |
| Improvement (change in HFMSE 1-2 points), n (%) | 26 (36) | 17 (26) | 12 (18) | 16 (25) | 12 (21) | 10 (23) | 4 (14) |
| Clinically meaningful improvement (change in HFMSE ≥3), n (%) | 26 (36) | 35 (53) | 40 (62) | 38 (60) | 35 (63) | 26 (60) | 20 (71) |
| Any improvement (change in HFMSE ≥1), n (%) | 52 (72) | 52 (79) | 52 (80) | 54 (86) | 47 (84) | 36 (84) | 24 (86) |

*One patient with SMA2 was not assessed at T6 but was assessed at the next 4 time points.
