# Supplementary material for: Long-term nusinersen treatment across a wide spectrum of spinal muscular atrophy severity: a real-world experience
Source: Orphanet J Rare Dis. 2023 Aug 4;18:230. doi: 10.1186/s13023-023-02769-4 (PMC10401775; doi:10.1186/s13023-023-02769-4)
Supplement: Supplementary file 3 — Additional file 3: Changes versus baseline (T0) in SMA3 patients (n = 67) who were assessed by the Hammersmith Functional Rating Scale Expanded (HFMSE), including 48 ambulant and 19 non-ambulant patients. [file 13023_2023_2769_MOESM3_ESM.docx]

**Additional file 3.**  Changes versus baseline (T0) in SMA3 patients (n=67) who were assessed by the Hammersmith Functional Rating Scale Expanded (HFMSE), including 48 ambulant and 19 non-ambulant patients.

| **Changes vs T0 in HFMSE for SMA3** | **Month of treatment (no. of patients)** | | | | | | |
| --- | --- | --- | --- | --- | --- | --- | --- |
|  | **T6 (67)** | **T10 (60)** | **T14 (59)** | **T18 (57)** | **T22 (50)** | **T26 (38)** | **T30 (25)** |
| Worsening (change in HFMSE <0), n (%) | 6 (9) | 1 (1.7) | 3 (5) | 3 (6) | 1 (2) | 1 (2.7) | 1 (4) |
| Stable (HFMSE = 0), n (%) | 13 (19) | 12 (20) | 8 (14) | 5 (9) | 6 (12) | 5 (13) | 2 (8) |
| Improvement (change in HFMSE 1-2 points), n (%) | 23 (34) | 15 (25) | 11 (19) | 14 (25) | 10 (20) | 8 (21) | 4 (16) |
| Clinically meaningful improvement (change in HFMSE ≥3), n (%) | 25 (37) | 32 (53) | 37 (63) | 35 (61) | 33 (66) | 24 (63) | 18 (72) |
| Any improvement (change in HFMSE ≥1), n (%) | 48 (72) | 47 (78) | 48 (81) | 49 (86) | 43 (86) | 32 (84) | 22 (88) |
