# Supplementary material for: Long-term nusinersen treatment across a wide spectrum of spinal muscular atrophy severity: a real-world experience
Source: Orphanet J Rare Dis. 2023 Aug 4;18:230. doi: 10.1186/s13023-023-02769-4 (PMC10401775; doi:10.1186/s13023-023-02769-4)

Additional file 4. Changes in HFMSE score from T0 (baseline) to T6 (6 months)[A],T10 (10 months)[B], T14 (14 months) [C], T18 (18 months) [D], T22 (22 months) [E], T26 (26 months) [F], T30 (30 months) [G] Each bar represents a single patient. HFMSE=Hammersmith Functional Motor Scale;

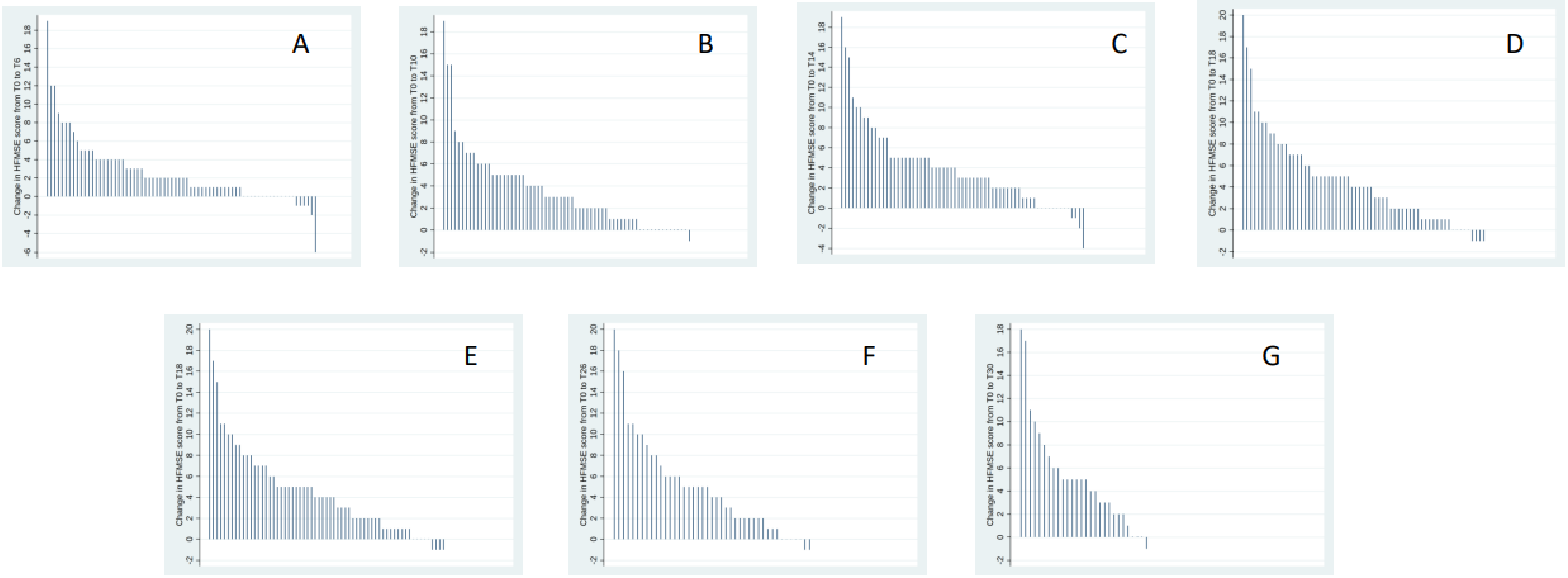

Supplement: Supplementary file 4 — Additional file 4: Changes in HFMSE score from T0 (baseline) to T6 (6 months)[A], T10 (10 months)[B], T14 (14 months) [C], T18 (18 months) [D], T22 (22 months) [E], T26 (26 months) [F], T30 (30 months) [G]. Each bar represents a single patient. HFMSE = Hammersmith Functional Motor Scale. [file 13023_2023_2769_MOESM4_ESM.pdf]
