# Supplementary material for: Long-term nusinersen treatment across a wide spectrum of spinal muscular atrophy severity: a real-world experience
Source: Orphanet J Rare Dis. 2023 Aug 4;18:230. doi: 10.1186/s13023-023-02769-4 (PMC10401775; doi:10.1186/s13023-023-02769-4)
Supplement: Supplementary file 5 — Additional file 5: Differences in mean scores between subsequent treatment time points in all functional tests: HFMSE, CHOP-INTEND, RULM, 6MWT. [file 13023_2023_2769_MOESM5_ESM.docx]

**Additional file 5.**  Differences in mean scores between subsequent treatment time points in all functional test**s: HFMSE, CHOP-INTEND, RULM, 6MWT**

|  |  | **Compared time points of treatment** | | | | | | |
| --- | --- | --- | --- | --- | --- | --- | --- | --- |
| **HFMSE** |  | **T6 vs**  **T0** | **T10 vs**  **T6** | **T14 vs**  **T10** | **T18 vs**  **T14** | **T22 vs T18** | **T26 vs T22** | **T30 vs**  **T26** |
|  | Difference in mean score | 2.53 | 1.06 | 0.53 | 0.43 | 0.27 | 0.33 | 0.33 |
|  | p value* | **<0.001** | **<0.001** | **<0.001** | **<0.001** | **0.007** | **0.011** | **0.011** |
| **CHOP-INTEND** | Difference in mean score | 2.23 | 0.95 | 0.79 | 0.94 | 0.65 | 0.65 | 0.8 |
|  | p value** | **<0.001** | **<0.001** | **0.003** | **<0.001** | **<0.001** | 0.109 **and 0.043** | 0.25 and **0.05** (only 5 patients) |
| **RULM** | Difference in mean score | 0.6 | 0.98 | 0.19 | 0.48 | 0.13 | 0.24 | 0.35 |
|  | p value*** | **0.047** | **<0.001** | 0.142 | **0.004** | 0.374 | 0.121 | **0.035** |
| **6MWT** | Difference in mean distance | 5.4 | 11.1 | -3.5 | -5.7 | -5.8 | 13.1 | 5.6 |
|  |  |  |  |  |  |  |  |  |
|  |  |  |  |  |  |  |  |  |
|  |  |  |  |  |  |  |  |  |
|  | p value **** | 0.288 | 0.094 | 0.710 | 0.834 | 0.341 | 0.132 | 0.636 |

*p value was assessed by the Wilcoxon and Student-t tests, and the results were the same (refers to HFMSE)**Wilcoxon and Student-t tests; the results were the same except for T26 vs T22 (Wilcoxon test 0.109; student test p=0.043) and for T30 vs T26 (Wilcoxon test, 0.25; Student-t test, p=0.05) (refers to CHOP-INTEND)*** p value was assessed by the Wilcoxon and Student-t tests, and the results were the same (refers to RULM)**** p value was assessed by the Wilcoxon and Student-t tests, and the results were the same (refer to 6MWT)
