# Supplementary material for: Long-term nusinersen treatment across a wide spectrum of spinal muscular atrophy severity: a real-world experience
Source: Orphanet J Rare Dis. 2023 Aug 4;18:230. doi: 10.1186/s13023-023-02769-4 (PMC10401775; doi:10.1186/s13023-023-02769-4)
Supplement: Supplementary file 6 — Additional file 6: Changes in the HFMSE score versus baseline (T0) for ambulant and non-ambulant patients and comparison the results. [file 13023_2023_2769_MOESM6_ESM.docx]

**Additional file 6.** Changes in the HFMSE score versus baseline (T0) for ambulant and non-ambulant patients and comparison the results

| **Changes in HFMSE HFMSE for ambulant and non-ambulant patients** | **Month of treatment** | | | | | | |
| --- | --- | --- | --- | --- | --- | --- | --- |
|  | **T6** | **T10** | **T14** | **T18** | **T22** | **T26** | **T30** |
| **Ambulant n=48 at T0**  Mean score (median, SD, min-max) | 2.42  (2, 3.2, -6–12) | 3.64  (3, 2.9, 0-15) | 4.07  (3.5, 3.6, -4–16) | 4.55  (4.5, 3.8, -1–17) | 4.75  (5, 3.9,  -4–17) | 4.64  (5, 4.2,  -1–18) | 4.45  (4.5, 4.1,  -1–18) |
| **number of ambulant patients** | 48 | 44 | 42 | 40 | 36 | 28 | 20 |
| **p value** | **<0.001** | **<0.001** | **<0.001** | **<0.001** | **<0.001** | **<0.001** | **<0.001** |
| **Non-ambulant n=25 at T0**  Mean score (median, SD, min-max) | 2.75  (1.5, 4.3, -1–19) | 3.48  (1.5, 5.1, -1–19) | 3.78  (3, 5.1, -2–19) | 4.35  (3, 5.2, -1–20) | 4.55  (5, 3.9,  -4–17) | 5.67  (4, 6.1,  -1–20) | 7  (6.5, 5.5,  0–17) |
| **number of non-ambulant patients** | 24 | 22 | 23 | 23 | 20 | 15 | 8 |
| **p value** | **<0.001** | **<0.001** | **<0.001** | **<0.001** | **<0.001** | **0.001** | **0.016** |
| Difference in mean  **ambulant versus non-ambulant patients**  mean (95% CI) | -0.33  (-1.5-2.1) | 0.16  (-1.8-2.1) | 0.29  (-2.5-1.9) | 0.2  (-2.5-2.1) | 0.2  (-2.7-2.3) | -1.03  (-2.1-4.2) | -2.55  (-1.4-6.5) |
| **p value** | 0.731 | 0.345 | 0.285 | 0.423 | 0.363 | 0.924 | 0.248 |
