# Supplementary material for: Long-term nusinersen treatment across a wide spectrum of spinal muscular atrophy severity: a real-world experience
Source: Orphanet J Rare Dis. 2023 Aug 4;18:230. doi: 10.1186/s13023-023-02769-4 (PMC10401775; doi:10.1186/s13023-023-02769-4)
Supplement: Supplementary file 7 — Additional file 7: Changes versus baseline (T0) in SMA1 patients (n = 9) who were assessed by the CHOP-INTEND N -number of patients; (%) percentage of patients. [file 13023_2023_2769_MOESM7_ESM.docx]

**Additional file 7.** Changes versus baseline (T0) in SMA1 patients (n=9) who were assessed by the CHOP-INTEND N -number of patients; (%) percentage of patients.

| **Changes vs T0 in CHOP-INTEND for SMA1 patients** | **Month of treatment (no. of patients)** | | | | | | |
| --- | --- | --- | --- | --- | --- | --- | --- |
|  | **T6**  **(9)*** | **T10 (9)** | **T14 (8)** | **T18**  **(8)** | **T22**  **(8)** | **T26**  **(8)** | **T30**  **(4)** |
| Worsening (change in CHOP-INTEND <0), n (%) | 1 (11) | 1 (11) | 0 (0) | 0 (0) | 0 (0) | 0 (0) | 0 (0) |
| Stable (CHOP-INTEND = 0), n (%) | 3 (33) | 2 (22) | 2  (25) | 2 (25) | 1 (12.5) | 0 (0) | 0 (0) |
| Improvement (change in CHOP-INTEND = 1-3), n (%) | 4  (44) | 2 (22) | 2  (25) | 1 (12.5) | 2 (25) | 1 (12.5) | 0 (0) |
| Clinically meaningful improvement (change in CHOP-INTEND  ≥4 ), n (%) | 1 (11) | 4 (44) | 4 (50) | 5 (62.5) | 5 (62.5) | 7 (87.5) | 4  (100) |
| Any improvement (change in CHOP-INTEND ≥1), n (%) | 5 (56) | 6 (67) | 6 (75) | 6  (75) | 7 (87.5) | 8 (100) | 4  (100) |

*3 adult SMA1 patients who were not assessed at T0 were not included.
