# Supplementary material for: Long-term nusinersen treatment across a wide spectrum of spinal muscular atrophy severity: a real-world experience
Source: Orphanet J Rare Dis. 2023 Aug 4;18:230. doi: 10.1186/s13023-023-02769-4 (PMC10401775; doi:10.1186/s13023-023-02769-4)
Supplement: Supplementary file 8 — Additional file 8: Changes versus baseline (T0) in all patients (n = 44) who were assessed by the Children’s Hospital of Philadelphia Infant Test of Neuromuscular Disorders (CHOP-INTEND).SMA1c (n = 9; additional 3 patients started evaluation in the study at T10, T14, and T18, respectively), SMA2 (n = 13), SMA3 (n = 22) patients. [file 13023_2023_2769_MOESM8_ESM.docx]

**Additional file 8**. Changes versus baseline (T0) in all patients (n=44) who were assessed by the Children's Hospital of Philadelphia Infant Test of Neuromuscular Disorders (CHOP-INTEND).SMA1(n=9; additional 3 patients started evaluation in the study at T10, T14, and T18, respectively), SMA2(n=13), SMA3 (n=22) patients.

| **Changes vs T0 in CHOP-INTEND for all 44 patients** | **Month of treatment (no. of patients)** | | | | | | |
| --- | --- | --- | --- | --- | --- | --- | --- |
|  | **T6**  **(44)** | **T10 (41)** | **T14**  **(38)** | **T18**  **(37)** | **T22 (26)** | **T26**  **(17)** | **T30 (5)** |
| Worsening (change in CHOP-INTEND <0), n (%) | 1 (2.3) | 1 (2) | 0 (0) | 0 (0) | 0 (0) | 1 (6) | 1(20) |
| Stable (CHOP-INTEND = 0), n (%) | 9 (20.5) | 6 (15) | 4 (10.5) | 4 (11) | 2 (8) | 0 (0) | 0 (0) |
| Improvement (change in CHOP-INTEND = 1-3), n (%) | 25 (57) | 19 (46) | 15 (39.5) | 10 (27) | 8 (31) | 5 (29) | 0 (0) |
| Clinically meaningful improvement (change in CHOP-INTEND  ≥4 ), n (%) | 9 (20.5) | 15 (37) | 19 (50) | 23 (62) | 16 (62) | 11 (65) | 4 (80) |
| Any improvement (change in CHOP-INTEND ≥1), n (%) | 34 (77.3) | 34 (83) | 34 (89.5) | 33 (87) | 24 (92) | 16 (94) | 4 (80) |
