# Supplementary material for: Long-term nusinersen treatment across a wide spectrum of spinal muscular atrophy severity: a real-world experience
Source: Orphanet J Rare Dis. 2023 Aug 4;18:230. doi: 10.1186/s13023-023-02769-4 (PMC10401775; doi:10.1186/s13023-023-02769-4)
Supplement: Supplementary file 9 — Additional file 9: Changes versus baseline (T0) in SMA2 patients (n = 13) who were assessed by the CHOP-INTEND. [file 13023_2023_2769_MOESM9_ESM.docx]

**Additional file 9.** Changes versus baseline (T0) in SMA2 patients (n=13) who were assessed by the CHOP-INTEND

| **Changes vs T0 in CHOP-INTEND for SMA2 patients** | **Month of treatment (no. of patients)** | | | | | | |
| --- | --- | --- | --- | --- | --- | --- | --- |
|  | **T6 (13)** | **T10 (11)** | **T14 (11)** | **T18**  **(11)** | **T22**  **(8)** | **T26 (5)** | **T30 (1)** |
| Worsening (change in CHOP-INTEND <0), n (%) | 0  (0) | 0  (0) | 0  (0) | 0  (0) | 0  (0) | 1  (20) | 1  (100) |
| Stable (CHOP-INTEND = 0), n (%) | 4  (31) | 3  (27) | 2  (18) | 2  (18) | 1 (12.5) | 0  (0) | 0  (0) |
| Improvement (change in CHOP-INTEND = 1-3), n (%) | 6  (46) | 5 (45.5) | 5 (45.5) | 4 (36.5) | 4  (50) | 3 (60) | 0 (0) |
| Clinically meaningful improvement (change in CHOP-INTEND  ≥4 ), n (%) | 3  (23) | 3  (27) | 4 (36.5) | 5 (45.5) | 3  (37.5) | 1 (20) | 0  (0) |
| Any improvement (change in CHOP-INTEND ≥1), n (%) | 9  (69) | 8 (73) | 9  (82) | 9 (82) | 7 (87.5) | 4  (80) | 0  (0) |
