# Supplementary material for: Long-term nusinersen treatment across a wide spectrum of spinal muscular atrophy severity: a real-world experience
Source: Orphanet J Rare Dis. 2023 Aug 4;18:230. doi: 10.1186/s13023-023-02769-4 (PMC10401775; doi:10.1186/s13023-023-02769-4)
Supplement: Supplementary file 10 — Additional file 10: Changes versus baseline (T0) in SMA3 patients (n = 22) who were assessed by CHOP-INTEND. [file 13023_2023_2769_MOESM10_ESM.docx]

**Additional file 10.** Changes versus baseline (T0) in SMA3 patients (n=22) who were assessed by CHOP-INTEND.

| **Changes vs T0 in CHOP-INTEND for SMA3 patients** | **Month of treatment (no. of patients)** | | | | | | |
| --- | --- | --- | --- | --- | --- | --- | --- |
|  | **T6**  **(22)** | **T10 (21)** | **T14 (19)** | **T18**  **(18)** | **T22 (10)** | **T26**  **(4)** | **T30 (0)** |
| Worsening (change in CHOP-INTEND <0), n (%) | 0 (0) | 0 (0) | 0 (0) | 0 (0) | 0 (0) | 0 (0) | NA |
| Stable (CHOP-INTEND = 0), n (%) | 2  (9) | 1 (4.5) | 0 | 0 | 0 | 0 | NA |
| Improvement (change in CHOP-INTEND = 1-3), n (%) | 15 (68) | 12  (57) | 8  (42) | 5  (28) | 2  (20) | 1  (25) | NA |
| Clinically meaningful improvement (change in CHOP-INTEND  ≥4 ), n (%) | 5 (23) | 8 (38) | 11 (58) | 13 (72) | 8  (80) | 3  (75) | NA |
| Any improvement (change in CHOP-INTEND ≥1), n (%) | 20 (91) | 20 (95) | 19  (100) | 18  (100) | 10 (100) | 4  (100) | NA |
