# Supplementary material for: Long-term nusinersen treatment across a wide spectrum of spinal muscular atrophy severity: a real-world experience
Source: Orphanet J Rare Dis. 2023 Aug 4;18:230. doi: 10.1186/s13023-023-02769-4 (PMC10401775; doi:10.1186/s13023-023-02769-4)
Supplement: Supplementary file 11 — Additional file 11 Changes versus baseline (T0) in 51 patients (9 with SMA2 and 43 with SMA3) assessed by Revised Upper Limb Module (RULM); n = number of patients; (%) percentage of patients. [file 13023_2023_2769_MOESM11_ESM.docx]

**Additional file 11**  Changes versus baseline (T0) in 51 patients (9 with SMA2 and 43 with SMA3) assessed by Revised Upper Limb Module (RULM); n=number of patients; (%) percentage of patients

| **Changes vs T0 in RULM for 51 patients** | **Month of treatment (no. of patients)** | | | | | | |
| --- | --- | --- | --- | --- | --- | --- | --- |
|  | **T6**  **(20)** | **T10 (20)** | **T14**  **(34)** | **T18**  **(43)** | **T22**  **(37)** | **T26**  **(34)** | **T30**  **(23)** |
| Worsening (change in RULM <0), n (%) | 1  (5) | 3 (15) | 3  (9) | 6  (14) | 5  (13.5) | 4  (12) | 1  (4) |
| Stable (change in RULM = 0), n (%) | 13  (65) | 11 (55) | 14  (41) | 14  (33) | 11  (30) | 10  (29) | 8  (35) |
| Improvement (change in RULM = 1), n (%) | 1 (5) | 4 (20) | 8  (24) | 12  (28) | 13 (35) | 7  (20.5) | 4  (17) |
| Clinically meaningful improvement (change in RULM ≥2), n (%) | 5 (25) | 2 (10) | 9 (26.5) | 11  (25.6) | 8 (22) | 13 (38) | 10  (43.5) |
| Any improvement (change in RULM ≥1), n (%) | 6 (30) | 6 (30) | 17 (50) | 23 (53.5) | 21 (57) | 20  (59) | 14 (61) |
