# Supplementary material for: Long-term nusinersen treatment across a wide spectrum of spinal muscular atrophy severity: a real-world experience
Source: Orphanet J Rare Dis. 2023 Aug 4;18:230. doi: 10.1186/s13023-023-02769-4 (PMC10401775; doi:10.1186/s13023-023-02769-4)
Supplement: Supplementary file 13 — Additional file 13: Changes in 6 min walk test (6MWT) results versus baseline (T0). [file 13023_2023_2769_MOESM13_ESM.docx]

**Additional file 13.** Changes in 6 minute walk test (6MWT) results versus baseline (T0)

| **Changes in 6MWT vs T0** | **Month of treatment (no. of patients)** | | | | | | |
| --- | --- | --- | --- | --- | --- | --- | --- |
|  | **T6**  **(15)** | **T10**  **(14)** | **T14 (19)** | **T18 (22)** | **T22 (18)** | **T26 (16)** | **T30 (12)** |
| Worsening (change in 6MWT <0), n (%) | 6 (40) | 2(14) | 5 (26) | 4 (18) | 9 (50) | 4 (25) | 4 (33) |
| No change, n (%) | 0 | 1 (7) | 0 | 1(4.5) | 0 | 1 (6) | 0 |
| Small improvement (6MWT >0 <30 m), n (%) | 4 (27) | 7 (50) | 9(47) | 10(46) | 2(11) | 4 (25) | 2 (17) |
| Clinically meaningful improvement (change in 6MWT ≥30 m), n (%) | 5 (33) | 4(29) | 5 (26) | 7 (32) | 7 (39) | 7 (44) | 6 (50) |
| Any improvement (change 6MWT >0), n (%) | 9 (60) | 11(79) | 14 (74) | 17(77) | 9 (50) | 11(69) | 8 (67) |
