# Supplementary material for: Long-term nusinersen treatment across a wide spectrum of spinal muscular atrophy severity: a real-world experience
Source: Orphanet J Rare Dis. 2023 Aug 4;18:230. doi: 10.1186/s13023-023-02769-4 (PMC10401775; doi:10.1186/s13023-023-02769-4)
Supplement: Supplementary file 14 — Additional file 14: Results of the multivariate regression (dependent variable: HFMSE score versus baseline-T0). [file 13023_2023_2769_MOESM14_ESM.docx]

**Additional file 14.** Results of the multivariate regression (dependent variable: HFMSE score versus baseline-T0)

| Time points | T6 | T10 | T14 | T18 | T22 | T26 | T30 |
| --- | --- | --- | --- | --- | --- | --- | --- |
| Sex | -1.815**  (0.857) | -2.505***  (0.936) | -1.794*  (1.067) | -1.611  (1.123) | -1.593  (1.270) | -2.316  (1.393) | -2.600  (1.651) |
| T0 | -0.029  (0.023) | -0.028  (0.025) | -0.029  (0.028) | -0.032  (0.029) | -0.040  (0.032) | -0.075**  (0.036) | -0.112**  (0.044) |
| BMI low | 0.639  (1.038) | 0.539  (1.142) | -0.168  (1.263) | -0.173  (1.322) | -0.490  (1.406) | -2.880*  (1.626) | -0.740  (1.952) |
| Constant | 4.475***  (1.046) | 5.937***  (1.102) | 6.080***  (1.243) | 6.564***  (1.291) | 7.186***  (1.509) | 9.674***  (1.665) | 11.01***  (2.007) |
| R2 | 0.086 | 0.122 | 0.065 | 0.057 | 0.061 | 0.222 | 0.297 |
| N | 72 | 66 | 65 | 63 | 56 | 43 | 28 |

Notes: the standard errors of the coefficients are given in parentheses. The symbols *, **, and *** denote statistical significance at the 0.1, 0.05, and 0.01 level, respectively
