# Supplementary material for: Long-term nusinersen treatment across a wide spectrum of spinal muscular atrophy severity: a real-world experience
Source: Orphanet J Rare Dis. 2023 Aug 4;18:230. doi: 10.1186/s13023-023-02769-4 (PMC10401775; doi:10.1186/s13023-023-02769-4)
Supplement: Supplementary file 15 — Additional file 15: Results for Patient Global Impression – Improvement (PGI-I) assessment at subsequent time points of treatment: data for 120 all patients. [file 13023_2023_2769_MOESM15_ESM.docx]

**Additional file 15.** Results for **Patient Global Impression – Improvement (PGI-I)** assessment at subsequent time points of treatment: data for 120 all patients**.**

| **PGI-I response at each time point of treatment** | **Month of treatment (no. of patients)** | | | | | | |
| --- | --- | --- | --- | --- | --- | --- | --- |
|  | **T6**  **(120)** | **T10**  **(116)** | **T14**  **(110)** | **T18**  **(104)** | **T22**  **(89)** | **T26 (64)** | **T30**  **(47)** |
| Very much improved | 1 (0.83) | 3 (2.6) | 1 (0.9) | 4 (4) | 2 (2.2) | 2 (3) | 1 (2) |
| Much improved | 16 (13) | 30 (26) | 26 (24) | 24 (23) | 30 (34) | 25 (39) | 23 (49) |
| Minimally improved | 78 (65) | 57 (49) | 55 (50) | 50 (48) | 36 (40.5) | 29 (45) | 16 (34) |
| Any improvement (PGI-I 1+2+3) | 94 (79) | 90 (78) | 82 (75) | 78 (75) | 68 (76) | 56 (87.5) | 40 (85) |
| No change | 23 (19) | 23 (21) | 23 (21) | 25 (24) | 19 (21) | 6 (9) | 7 (15) |
| Minimally worse | 2 (1.67) | 3 (2.6) | 5 (4.6) | 1 (1) | 2 (2.2) | 2 (3) | 0 (0) |
| Much worse | 0 (0) | 0 (0) | 0 (0) | 0 (0) | 0 (0) | 0 (0) | 0 (0) |
| Very much worse | 0 (0) | 0 (0) | 0 (0) | 0 (0) | 0 (0) | 0 (0) | 0 (0) |

Data are presented as number (%) of patients.
