# Supplementary material for: Long-term nusinersen treatment across a wide spectrum of spinal muscular atrophy severity: a real-world experience
Source: Orphanet J Rare Dis. 2023 Aug 4;18:230. doi: 10.1186/s13023-023-02769-4 (PMC10401775; doi:10.1186/s13023-023-02769-4)
Supplement: Supplementary file 16 [file 13023_2023_2769_MOESM12_ESM.odt]

Additional file 12. Changes in the RULM score  versus baseline for ambulant and non-ambulant patients and comparison the results

| Changes in RULM | Month of treatment |
| --- | --- |
| T6 | T10 | T14 | T18 | T22 | T26 | T30 |
| Ambulant n=26 at T0 Mean score (median, SD, min-max) | 0.25 (0, 0.7, 0–2) | 0.3 (0, 0.7,  0-2) | 0.63 (0, 1.5, 0-6) | 0.48 (0, 1.3, -2–5) | 0.5 (0, 1.2, -1–5) | 0.6 (0, 1.5, -3–4) | 1.13 (1, 1.6, -1–5) |
| N | 8 | 10 | 16 | 23 | 36 | 20 | 15 |
| p value | 0.008 | 0.004 | <0.001 | <0.001 | <0.001 | <0.001 | <0.001 |
| Non-ambulant n=25 at T0 Mean score (median, SD, min-max) | 0.83 (0, 1.34, -1–3) | 0.3 (0, 1.6,  -2–4) | 0.94 (1, 1.8,  -4–4) | 1.40 (1, 2.3, -2–6) | 1.82 (1, 2.6, -2–8) | 2.35 (2, 2.4, -1–7) | 3.5 (4, 3, 0–8) |
| N | 12 | 10 | 18 | 20 | 20 | 14 | 8 |
| p value | 0.183 | 0.625 | 0.475 | 0.145 | 0.069 | 0.001 | 0.014 |
| Difference in mean ambulant versus non-ambulant patients mean (95% CI) | -0.58 (-1.6-0.5) | 0 (-1.2-1.2) | -0.31 (-1.5-0.8) | -0.92 (-2.1–0.2) | -1.32 (-2.6-0.0) | -1.75 (-3.1-0.4) | -2.37 (-4.3-0.4) |
| p value | 0.381 | 0.889 | 0.008 | 0.036 | 0.033 | 0.023 | 0.067 |

N =number of patients at each time point of treatment
